# Primary Health Care in context with the plurality in the care of people with tuberculosis

**DOI:** 10.1590/1518-8345.6978.4473

**Published:** 2025-02-17

**Authors:** Edna Ferreira Santos, Fátima Teresinha Scarparo Cunha, Pedro Fredemir Palha, Afranio Lineu Kritski

**Affiliations:** 1 Universidade Federal do Rio de Janeiro, Faculdade de Medicina, Rio de Janeiro, RJ, Brazil; 2 Secretária Municipal de Saúde, Superintendência de Atenção Primária, Coordenação de Doenças Crônicas Não Transmissíveis, Rio de Janeiro, RJ, Brazil; 3 Universidade Federal do Estado do Rio de Janeiro, Escola de Enfermagem Alfredo Pinto, Rio de Janeiro, RJ, Brazil; 4 Universidade de São Paulo, Escola de Enfermagem de Ribeirão Preto, PAHO/WHO Collaborating Centre for Nursing Research Development, Ribeirão Preto, SP, Brazil

**Keywords:** Public Policy, Primary Health Care, Semantics, Tuberculosis, Health Services, Delivery of Health Care

## Abstract

**Objective:**

to analyze the plurality of care provided to individuals with tuberculosis in Primary Health Care services in Rio de Janeiro, RJ, Brazil.

**Method:**

this qualitative study was conducted from 2017 to 2021. Discourse Analysis was adopted as the theoretical-methodological framework. Semi-structured interviews were conducted with service managers, healthcare professionals, and patients. The interviews were categorized using ATLAS.ti 8. The theoretical contributions of public space, plurality, and natality, as discussed by Hannah Arendt, were used to anchor the Discursive Formations on the health field.

**Results:**

the discourses revealed the complexity and challenges of organizing and implementing care practices among individuals with tuberculosis, highlighting the relevance of considering the historical, social, and individual contexts of managers, patients, and healthcare professionals. Difficulties were found in the operationalization of Health Care Networks and in searching for the patients’ uniqueness.

**Conclusion:**

an ongoing dispute was found in the interactions between Primary Health Care and the National Tuberculosis Control Program, between policies that value plurality, organize Health Care Networks, and consider democratic rights in public spaces.

## Highlights

**(1)**Healthcare provided to individuals with tuberculosis should be considered from a plural perspective. **(1)**Changes in universal treatment favor patient-centered care. **(2)**The care provided for chronic diseases is complex and requires organization in thematic networks. **(3)**Universal systems organized in networks favor innovative care practices. **(4)**The participation and empowerment of individuals with illnesses improve health care delivery.

## Introduction

Primary Health Care (PHC) is the healthcare model that facilitates the implementation of the Unified Health System (SUS, acronym in Portuguese), as it implies changes in local management policies and in the organization of care practices^(1)^. In this sense, the current healthcare scenario has demanded significant changes for the management of infectious and acute conditions, as well as chronic diseases, through the implementation of effective strategies, which impose challenges on the organization of healthcare and from an economic perspective. The aim is to achieve better health indicators within healthcare systems, especially in the control of Tuberculosis (TB) in PHC.

Evidence indicates that strengthening local healthcare systems integrated into Health Care Networks (RAS, acronym in Portuguese) can support the integration of services and improve the quality of healthcare delivery^(2-3)^. The studies also highlight that comprehensive care, based on vertical and horizontal dimensions, ensures that patients can access the various points in the healthcare network; connects individual and collective clinical management; enhances referral and counter-referral mechanisms; improves the services’ efficiency and rationality; and leads to economies of scale and scope, contributing to improved access and the sustainability of the healthcare system.

We argue that cooperation, coordination, and regulation within networks relate to the notion of interdependence between healthcare professionals and services, serving as a tool to balance supply and demand, thereby promoting better effectiveness of health indicators. The importance of intersectoral collaboration within the social protection system for populations living in unfavorable living and health conditions and requiring income transfer policies to survive and maintain their treatment^(2-3)^, such as those affected by tuberculosis, is also emphasized.

Thus, the implementation of organizational guidelines for decentralization and regionalization, starting in the 1990s, promoted advancements and imposed challenges in the SUS’ organizational structure^(4)^. In this organizational arrangement, PHC is the center of communication, which can facilitate the interaction of individual and collective care practices from the perspective of health surveillance, within a single territory, based on institutional collaboration supported by local strengths^(1)^.

Regionalization gains visibility in the public sphere by putting pressure on political agendas, leading federal entities to take on the planning, management, and financing of health responsibilities. The objective is to organize a decentralized and regionalized system to heed the population’s health conditions, based on the territory and the flow of care services to compose RAS^(5)^.

This political process introduced new meanings and ways of occupying governance spaces, anchored in scientific evidence that points to the need to change these practices. In this regard, the organization of vertical programs, focused on the fragmentation of services and universalizing actions, makes it difficult to strengthen collaborative spaces and shift the organizational axis of the SUS towards the establishment of integrated planning with an emphasis on plurality^(4–10)^, a fact perceived in the TB care program.

Thus, the structuring model for TB control is designed through actions that connect from the national to the local level, incorporated into control as a standard unit to identify, care for, and connect patients to PHC^(6,8-9)^. This modeling of the healthcare system, consisting of several points of care, has been advancing since 2010, to meet the needs for longitudinal care and to ensure services mediated by the RAS for chronic communicable and non-communicable diseases^(2,4,8-9)^.

Thus, it is in this context of reorganization of the RAS that the treatment of TB, a communicable disease, shows a discrepancy between what is recommended by the National Tuberculosis Control Program (PNCT in Portuguese) and the PHC actions to monitor and care for TB.

Therefore, this study’s relevance lies in the need to advance the implementation of an organizational model that accommodates plurality in governance spaces, including different actors who share differences and show singularities, enabling the emergence of new aspects in TB care provided within the SUS. The introduction of this debate into the healthcare agenda welcomes the principles and guidelines of decentralization and regionalization, providing positive stability for their incorporation by managers, healthcare professionals, and patients^(5,7)^.

Thus, the following question emerges: how does PHC interact with the vertical structure of the PNCT, considering the plurality of local arrangements, an ensure changes in care practices?

This study aimed to analyze the plurality of care actions performed for individuals with TB within PHC in Rio de Janeiro, RJ, Brazil.

## Method

### Study design

This qualitative study was supported by the French school of Discourse Analysis (DA)^(11)^.

### Study setting

This study was conducted at the PHC General Coordination (CAP in Portuguese) 3.1, one of the ten CAPs of the Municipal Health Department (SMS in Portuguese) of Rio de Janeiro, RJ, Brazil. The population served in this area comprises 886,551 people, distributed across 28 neighborhoods, 50% of which are highly densely populated, exceeding 12,517 people per neighborhood, and the entire area contains 32 PHC units.

### Timeframe

Data collection occurred at two points in time: the second half of 2017 and the first half of 2021. This extended period was due to numerous complications, including the interviewees’ availability and the COVID-19 pandemic.

### Selection criteria

The participants were intentionally selected based on the following criteria: being a manager involved in the implementation of PHC, SMS, or CAP 3.1; being responsible for tuberculosis care within CAP 3.1; or being a physician or nurse with experience in the follow-up of individuals with TB, including those receiving treatment, discharged due to cure, or who abandoned treatment, in the territories of the PHC units.

### Participants

Fifteen participants were selected: eight men and seven women who identified as cisgender. The group included two managers, four doctors, four nurses, and five patients, aged between 18 and 65 years. The participants were identified by their positions to maintain confidentiality. CAP supported the selection process by assisting in identifying individuals who met the inclusion criteria, and they were contacted via email or WhatsApp; none refused to participate.

### Data collection instruments

The authors developed two semi-structured scripts containing questions directed at managers, healthcare professionals, and individuals with TB. The scripts focused on the participants’ understanding of the TB care policy and its implementation, the communication and bond established between workers and patients, and the patients’ self-care experiences. The interviews were audio-recorded and lasted between 20 and 60 minutes. Additionally, they were conducted at the PHC units or SMS locations chosen by the interviewees. There was no need for follow-up interviews.

### Data treatment and analysis

The ATLAS.ti 8 software was used to organize the empirical material. A single project, called the Hermeneutic Unit (HU), was created to include the interviews, which corresponded to the primary documents. The DA method was used for analysis, following three levels. The first stage, involving the transition from the linguistic surface to the discursive object, focused on analyzing beneath the surface by examining the text’s linguistic materiality - how it is said, who says it, and under what conditions. The second stage addressed the transition from the discursive object to the discursive process, aiming to understand the meanings produced in the first stage and delineate the discursive formations, based on the socio-historical context that determines what can and should be said. Finally, the third stage, which occurs concurrently with the second, involves outlining the discursive formation and its relationship with ideology. It examines how discursive formations differ from ideological formations, which are complex sets of attitudes and representations directly or indirectly linked to conflicting class positions^(11)^.

The four authors, all with experience in qualitative studies and public health, conducted the analyses together. Their academic and professional backgrounds enabled a detailed understanding of PNCT actions, both before and after its decentralization to PHC in Rio de Janeiro, RJ, Brazil. Data coding and the formulation of statements occurred interactively, allowing multiple perspectives and interpretations to be discussed and collectively agreed upon. The integration of each researcher’s reflections and field notes contributed to the development of complex discourses on the care provided to individuals with tuberculosis.

Discourse Analysis, as a theoretical-methodological framework, aims to place in the textual interludes an interpretation that reveals the meanings of discourse through its materiality, at the intersection of the historical and the linguistic^(12-16)^. It presents discourse as a symbolic object that moves within the participants’ narratives, producing meanings that enable the permanence, continuity, displacement, and transformation of individuals and their realities^(17-19)^.

The theoretical analysis within the DA framework follows the construction of a corpus designed to reveal how discourse functions in the production of meanings^(11,20)^. The discursive excerpts are fragments of the discursive situation, shaped by the conditions under which narratives are produced, both in immediate and broader contexts, and by the contextual interactions that involve the subject, situation, and memory^(20-23)^.

Thus, discourses are produced within the chain of historical events and processes of signification^(11,17,24)^, shaped both by the chronology of historical facts and by the conditions of production, which allow us to recognize the historicity within the text^(13,21-22)^. In this context, the conditions under which narratives are produced characterize the discursive process, making both paraphrase and polysemy visible as integral to Discursive Formations (DFs). It is from the heterogeneous and fluid nature of the participants’ positions that the possibility for the reorganization and movement of knowledge emerges^(25-27)^.

The contributions of this study lie in the understanding of DFs and the constant shifts in meaning that reveal the pre-constructed elements external to the discursive subject, defining influences that originate elsewhere, often without the individual’s awareness of this process. What has already been said, in another context, through discursive memory, is referred to as interdiscourse. It is through the interplay between what has already been said (interdiscourse) and the discourse in the act of being enunciated (intradiscourse) that the sayable is constituted^(13,17,19,23)^.

Thus, the concepts of interdiscourse, conditions of production, memory, ideology, and the paraphrastic and polysemic processes, which continually shift meaning between repetition and difference, were employed to bring forth the historicity of language, constituting the event of the text as discourse^(21,23,25-26)^. The discursive excerpts facilitated the mobilization of these concepts and their interaction with the theoretical-methodological framework, leading to the production of senses, the construction of the interpretive device, and the formation of three discursive blocks through the Discursive Sequences (DSs)^(11,20)^.

To anchor the DFs within the broader conditions of production^(28)^ in the field of public health policy, a dialogue was established with the theoretical framework of Hannah Arendt, who views the public space as a place for discussions, positions, and opinions, where relationships between individuals are formed^(7)^. In this space, individuals become actors, interact with one another, and communicate who they are to others^(29)^.

The central aspect of Arendtian politics lies in the very action that occurs when individuals act together, embracing spontaneity in their pursuit of distinction and singularity, which is inherently marked by plurality^(7,29-30)^. This is where the concept of natality emerges, understood as the act or effect of establishing new beginnings. It is based on the premise that every individual represents a new beginning, as they are born into a world that precedes them and will be transformed by them. Through action, they are also granted the ability to introduce something new into the world^(7,30-31)^.

It is important to note that the dimensions of plurality, public space, and natality^(7,30-32)^ are particularly relevant in this context, as they highlight that action involves a beginning that can lead to the introduction of something new into the world. Since this action is uncontrollable and unpredictable, its outcomes cannot be predetermined or foreseen. This perspective illuminates silenced issues, controversies, opacity, and tensions that are articulated, shifting the meanings of discourses circulating within the DFs^(33-35)^.

### Ethical aspects

This study was approved by the Institutional Review Board at SMS/RJ and the Federal University of Rio de Janeiro-UFRJ (opinion No. 4,332,298). All participants provided written informed consent. The confidentiality of the participants’ identities was ensured through coding.

## Results

The first stage explores how the managers’ discursive memories influence the repetition and vertical structure of the services and actions in TB treatment, highlighting how the conditions under which managers’ discourses were produced affect the organization of services and actions directed to the treatment of TB are organized.

The analysis of the discourses circulating among managers revealed linguistic marks that show contradictions and challenges. These marks appear in statements such as: *A bit police-like, let’s say... Almost, you know? Well, in my opinion, it continues to be... I don’t think it’s a matter of the Tuberculosis Program being normative, you know?* (M1). This discourse reflects the naturalization of the historical and normative practices of the PNCT, anchored in official documents from the Ministry of Health.

In this context, PHC plays a central role as a care provider, promoting an approach that is closer to the patients, mainly focusing on continuity and comprehensive care, as another manager states: *So, I think that, like, a lot of progress has been made, you know? Especially if we think about how restricted access was. Patients had to commute from distant places, without money. They had to come to a secondary unit, schedule an appointment... And the Family Health Team, they are there, you know? Practically inside the patients’ homes, you know? The patients practically live inside the Family Health Unit because the connection is very close* (M2). This statement shows the redefinition of public policy, highlighting advancements in the access to treatment through the proximity and accessibility provided by PHC.

The second stage makes us reflect on how discursive memory, associated with being a service user, can shape different trajectories in the production of life within the PHC context. In the case of the users of the healthcare system, this memory encompasses their experiences, narratives, and perceptions accumulated over time. It influences how individuals understand and interact with the healthcare system, shaping their expectations and actions.

This is particularly evident in the discourse of individuals in the position of users. One individual shares his experience with the tuberculosis treatment: *They won’t suspend us, because we can’t interrupt the treatment... It’s a big problem, but, like, today I manage to overcome every... setback in life because there are professionals who guide me. Nowadays, I understand a little bit about what tuberculosis is. It’s hard to deal with it... So imagine: a party... a dance... fun... beer. You having to take all that from your life. You stop drinking, it’s hard! ... It was the beginning of November when I started my treatment. The beginning of summer. Party. End of year celebrations. So it was... I had a lot of family friction with that* (P4). This discourse reveals the resistance to bureaucratic and depersonalized practices, highlighting the need for care to be adapted to the patients’ contexts of life.

The following DS shows how this discursive memory is intertwined with everyday experiences and challenges, revealing the plurality and ability of individuals to express themselves in public spaces based on their experiences and existences, pointing out the intersection between personal needs and public policies. It highlights how individual experiences are connected to broader contexts of health policies: *Everything involves the government, you know? Everything involves politics, right? And what happens? …So I think people need to value this more. They have to take this opportunity because death is no joke. Once you die, it’s over. Do you understand? While you’re alive, you have the opportunity to treat yourself, to take care of yourself… Certain situations come up… like in my case… I have four children, you know? Sometimes, like in my case, my house caught on fire… Today… I haven’t taken the medicine in 15 days. Do you understand? So since then, something happened… last night, I spent the whole night with a fever. My daughter also felt sick yesterday… Do you understand?* (P2).

P1’s discourse reveals the discursive memory of a SUS patient, in which obedience to medical instructions is constant*: I do what she says! I get there: “Hey, take the medicine!” And so on… I remember the day I started. It was on June 15th… But she says: “Hey, next week…” I think I’m going to change medicine today. I’m going to take another one… Yeah. I’m going to take another medicine that has two medications, if I’m not mistaken. So I do whatever she says!* (P1)*.* A regular pattern of behavior is evident in the context of the TB control program.

P5’s speech highlights the effects of meanings on the TB treatment trajectory, revealing both the difficulties the patients face when dealing with a chronic disease and the essential role PHC play in their recovery process: *Look, I’m being treated really well here. I take the medication every week. I’ve improved 100%. I got here weighing 43 kilograms. I have 53 and… 400 grams now! I weighed myself today. Look, that was a… an oversight on my part. I already had it for the first time and I… in a mistake I made last year, I thought the nurse had discharged me. But she hadn’t discharged me from the medication yet. So I abandoned the treatment!* (P5).

P3’s speech highlights the complexity of treating chronic diseases, emphasizing that recovery is not limited to prescribing and administering medication: *Yeah, because sometimes... Look, sometimes, there are a lot of people who don’t get better because there’s no one to help them... They think that all they have to do is come here, the doctor prescribes the medication, they take it... No. So if the person supports them and gives them strength, they can keep going...* (P3).

The third stage highlights how healthcare professionals’ memories are triggered to create meaningful discursive events. These discursive memories involve the repetition and displacement of experiences, characterized by deviations, failures, mistakes, and contradictions. These elements give meaning to the experiences of healthcare professionals when working in a public space, whose objective is to organize services and innovate health practices to address chronic conditions.

The conditions in which discourses are produced enable us to reflect on how discursive memories influence the way the physicians perceive and face challenges in their daily practices in the context of PHC*: So I think that in this sense, considering that we are not in the right conditions for Primary Health Care, I think the PNCT is becoming out of touch with reality. [Laughs.] It is becoming inadequate for the current reality of Primary Health Care. And this is thinking about Rio de Janeiro, where there is investment, you know?* (Ph4). This report highlights the disconnect between the PNCT and the reality of PHC in Rio de Janeiro, where there is investment, but even so, the conditions are not ideal.

This DS presents the importance of adjusting tuberculosis treatment, considering the temporality and bacillus transmission rate, as well as the patients’ social support network: *Yes, because of transmission, right? So we have to make sure that he takes it for the first 15 days, so that he doesn’t transmit it anymore, you know? And, depending on the case, we evaluate, right? Sometimes we release it in the second month, in the third month... weekly... If the person has a good support network, a family, you know?* (N3). This statement reflects how nurses’ memories and experiences guide their practices and decisions, shaping the way they face challenges in the treatment of chronic conditions such as tuberculosis.

In this next analysis, the physician’s speech addresses the fragmentation of care and decision-making based on a patient’s history, which goes beyond his/her presence in health services: *He started the treatment in prison, then he was cared for by a healthcare team, received some treatment, and then finished treatment with me. Only that he was also not taking the medication correctly, but we ended up discharging him because his sputum test was negative, and he had sort of completed the treatment period… We discharged him. Less than four months later he relapsed, came back, and then… Well, the beginning of the treatment was very good, but after approximately two months he started having the same problems...* (Ph3). Note how having knowledge of a patient’s previous history influences medical decisions, revealing difficulties in maintaining the continuity and effectiveness of treatment in fragmented contexts.

Another successful outcome was understanding how the process of decentralizing tuberculosis actions to the PHC units caused new interpretations and approaches to healthcare, influenced both by the professionals’ discourses and by the specific context in which they work: *I’ve been responsible for the line of care for a year and two months... Working on policy is complicated, and, with users, by talking about what policy means to them... So, when I arrived at the service to start this line of TB care plan, I first tried to see the territory* (N2).

This last DS acknowledges singularities in the context of PHC. The physician’s discourse, when embracing these singularities and affection, can lead the care process, breaking with traditional practices and enabling effective approaches, mediated by plurality: *I think we do not track patients very frequently. But those who are tracked and have a positive diagnosis… I think that from Primary Health Care, it makes all the difference in care, because it is a long treatment that requires a bond... It needs affection to understand what the limitations of care are... Understanding the peculiarities of each patient, of each family... Yes... I think that in this sense Primary Care can call the shots, you know?* (Ph2).

## Discussion

This study analyzed the inconsistencies between the PNCT standards and the need for a more plural approach in the care provided to people with tuberculosis within PHC, helping healthcare professionals and managers to identify the different meanings the population assigns to TB, promoting greater equity in health care.

In the first stage, the city’s management prioritizes the PNCT’s centralized policy, connecting the PHC units, as entities that perform fragmented actions through the Family Health Strategy teams^(8-9)^.

Considering the conditions in which discourses are produced, the managers’ discursive memories influence the organization of healthcare services. It is important to note that Rio de Janeiro still has a selective PHC that may be based on historical remnants^(4)^.

It is noteworthy that the DFs of the managers in the SUS of Rio de Janeiro revealed different interpretations, making invisible the tensions and differences experienced by professionals and patients in the face of a homogeneous discourse of universalizing practices. According to Arendt, the public space is essential for plurality, singularity and distinction. In this sense, the public space, in Arendt’s view, is the space par excellence of plural living, singularity and differences^(7)^.

The recognition of PHC as a communication center for RAS and coordinator of healthcare can bring plurality to institutional arrangements, improving access, equity and the humanization of care, contributing to the advancement of public health policies, especially in the fight against chronic diseases such as TB^(2,4)^.

Thus, the plural arrangements and managers’ discourses allow for questioning and problematizing the hegemony of TB treatment, connecting with other discourses in the territory where life happens^(23)^.

The discourses analyzed in the second stage reveal a variety of meanings surrounding the experience of patients receiving tuberculosis treatment, highlighting heterogeneous experiences^(35)^. The position of patients is constantly reinterpreted by the circulating discourses, enabling the individuals to experience the treatment in their unique way, often in disagreement with the standards imposed by health policies. These discourses also reflect the various forms of resistance to the treatment, showing that their actions and thoughts are shaped by interactions.

In this sense, studies emphasize the relevance of considering the experiences of individuals with TB and strengthening the relationship between patients and the healthcare staff to achieve successful treatment. Another aspect underscored by different studies is the importance of income transfer policies^(36-38)^ to ensure a satisfactory treatment.

Considering what TB represents to patients, the support provided by their families and the healthcare staff, along with social support, is essential^(39)^ for the treatment to be adequate, even though patients may feel that their space is being invaded^(37)^. Thus, studies highlight the need for healthcare professionals to continually reassess their activities and skills to provide holistic and comprehensive care^(36-37)^, from the perspective of patient-centered care.

From this perspective, plurality allows for rupturing the limits of control and creating new relationships within PHC, establishing a public space with the potential to establish the new^(7,30)^. Thus, timely communication in health care networks^(4)^ reveals that supported self-care is crucial for the individuals to participate and be co-responsible for their health.

However, the treatment’s side effects and TB’s social impact often lead patients to experience mental illnesses, like depression, which interfere with the treatment^(40)^. A lack of social and family support may lead to treatment abandonment in the presence of mental illness, potentially leading to various disorders. For this reason, ongoing education is essential for healthcare professionals to provide care to these patients^(37)^.

In the third stage, the professionals’ memory networks generated events^(23)^ that influenced their discourses on health care, suggesting the inclusion of plural practices. These discourses connect to the concepts of public space^(7)^ and natality^(30)^, revealing the ability to start something new amid complex human interactions.

In this discursive web, the professionals’ discourses reveal contradictions and willingness to recognize the singular individual with TB in the public space, circulating new memories. This openness can be considered by PHC as a key to dialogue linked to everyday events^(41)^. The living conditions of ill individuals, which give meaning to the struggle for life and prompt a review of standardized control, can lead to a reflection on the professionals’ ability to embrace the repercussions of a chronic disease^(6,9)^, such as TB, whose treatment id considerably long^(39)^.

Therefore, as TB is a public health concern that requires prolonged treatment, it cannot be addressed solely with policies that focus on ending treatment; neglecting social determinants may lead to treatment failure^(37-38,42)^.

In this sense, the PHC in the units in Rio de Janeiro addressed in this study challenges the authoritarian actions of the PNCT, which are based on hierarchical control. These actions use universal practices, recognized by managers, professionals and patients as a specific language of the program that authorizes its way of operating. Such an approach attributes a social value to the discourse on TB, reinforcing practices centered on treatment control and restricting relationships^(13)^.

This study enabled an understanding that there are different discursive matrices in the circulating discourses. Paraphrastic matrices close the discourse in a unilateral control, while polysemic matrices open the PHC discourse to autonomy through plurality. The presence of different and plural thoughts in public spaces can generate new discourses that express inclusive and participatory forms of care.

In this study, the path to breaking with structural control within PHC was found in the dialogue with Arendt’s ideas on public space, plurality, and natality. These ideas emerge from the discursive formations of patients and professionals^(30-31)^, promoting a more porous environment centered on individuals’ singularities.

The implications for the health field and professional teams include the possibility of promoting new beginnings and political action that recognizes TB as a chronic disease. This recognition facilitates the creation of new connections and practices based on the attributes of PHC, reclaiming the possibility of becoming healthy through plurality.

In this sense, research on the care provided to individuals with multidrug-resistant tuberculosis (MDR-TB) highlights the importance of a patient-centered approach, valuing the plurality of experiences. Patient-centered care enables professionals to establish and maintain meaningful bonds with patients, overcoming the limitations imposed by rigid protocols and standardized norms^(43)^.

Finally, this study’s limitations concern its convenience sample. Although the results allowed us to understand the perspective of patients with TB, no information was collected from family members or from close individuals comprising the social support network of individuals with TB. Therefore, future studies are suggested to include information from the participants’ social support network. Such a reflective approach can improve the quality of research and the relevance of findings to promote more effective and inclusive interventions in TB care.

## Conclusion

This study analyzed how tuberculosis (TB) control, characterized by a hierarchical and normative model, can erase the individuality of patients, rendering invisible feelings such as helplessness, anguish, isolation, and fear of death. The National Tuberculosis Control Program (PNCT), with its emphasis on standardized manuals, techniques, and a universal language, contrasts with the approach within Primary Health Care (PHC), which values the singularities of local contexts and realities where life unfolds.

There is an ongoing dispute between PHC’s plural policy— which values differences and organizes the Health Care Networks to ensure individuals’ right to health— and the PNCT’s normative model. PHC aims to acknowledge and address patients’ singularities, proposing patient-centered care tailored to the specificities of each territory.

Supporting the embryonic governance spaces resulting from the decentralization and regionalization of health is essential to strengthen this plural and democratic approach. These spaces should promote the leadership of local actors, linking them to the plurality of patient-centered care. This strategy can more effectively ensure the democratic right to health, meeting the specific needs of patients and promoting more humane and effective care.
