# Supplementary material for: Primary Health Care in context with the plurality in the care of people with tuberculosis
Source: Rev Lat Am Enfermagem. 2025 Feb 17;33:e4473. doi: 10.1590/1518-8345.6978.4473 (PMC11835005; doi:10.1590/1518-8345.6978.4473)
Supplement: Translation

# Translation: A atenção primária em contexto com a pluralidade no cuidado de pessoas com tuberculose

**DOI:** 10.1590/1518-8345.6978.4474

**Authors:** Edna Ferreira Santos, Fátima Teresinha Scarparo Cunha, Pedro Fredemir Palha, Afranio Lineu Kritski

**Affiliations:** 1 Universidade Federal do Rio de Janeiro, Faculdade de Medicina, Rio de Janeiro, RJ, Brasil; 2 Secretária Municipal de Saúde, Superintendência de Atenção Primária, Coordenação de Doenças Crônicas Não Transmissíveis, Rio de Janeiro, RJ, Brasil; 3 Universidade Federal do Estado do Rio de Janeiro, Escola de Enfermagem Alfredo Pinto, Rio de Janeiro, RJ, Brasil; 4 Universidade de São Paulo, Escola de Enfermagem de Ribeirão Preto, Centro Colaborador da OPAS/OMS para o Desenvolvimento da Pesquisa em Enfermagem, Ribeirão Preto, SP, Brasil

**Keywords:** Política Pública, Atenção Primária à Saúde, Semântica, Tuberculose, Serviços de Saúde, Atenção à Saúde

## Abstract

**Objetivo:**

analisar a pluralidade do cuidado de pessoas adoecidas por tuberculose na Atenção Primária à Saúde no município do Rio de Janeiro.

**Método:**

trata-se de estudo qualitativo, realizado de 2017 a 2021, utilizou-se a Análise de Discurso como referencial teórico-metodológico. Foram coletados dados de gestores, profissionais de saúde e usuários por meio de entrevistas semiestruturadas, categorizadas com o *software* ATLAS.ti 8. Para ancorar as Formações Discursivas, no campo da saúde, dialogou-se com os aportes teóricos de espaço público, pluralidade e natalidade, discutidos por Hannah Arendt.

**Resultados:**

os discursos analisados revelam a complexidade e os desafios na organização e execução das práticas de cuidado à pessoa adoecida com tuberculose, destacando a importância do contexto histórico, social e individual de gestores, usuários e profissionais de saúde. Identificaram-se desafios na operacionalização das Redes de Atenção à Saúde e na busca pela singularidade dos usuários.

**Conclusão:**

nas interações entre a Atenção Primária à Saúde e o Programa Nacional de Controle de Tuberculose há uma constante disputa entre as políticas que valorizam a pluralidade, organizam as Redes de Atenção à Saúde e consideram o direito democrático, nos espaços públicos.

## Destaques

**(1)** O cuidado de pessoas com tuberculose deve ser considerado a partir da pluralidade. **(2)** Mudanças no tratamento universalizante favorecem o cuidado centrado no usuário. **(3)** O cuidado para doenças crônicas é complexo e requer organização em redes temáticas. **(4)** Sistemas universais organizados em redes favorecem práticas inovadoras no cuidado. **(5)** A participação e emancipação dos sujeitos adoecidos potencializa o cuidado em saúde.

## Introdução

O modelo de atenção que capilariza a implementação do Sistema Único de Saúde (SUS) é a Atenção Primária à Saúde (APS), por implicar mudanças nas políticas locais de gestão e organização das práticas de atenção^(1)^. Nesse sentido, o cenário sanitário atual tem exigido mudanças importantes, tanto para o manejo das condições infecciosas e agudas quanto das crônicas, por meio de estratégias eficazes, constituindo-se em um desafio para a perspectiva econômica e a organização da atenção à saúde. Considerando a obtenção de melhores indicadores sanitários junto aos sistemas de saúde^(2)^, em especial, para o controle da Tuberculose (TB) na APS.

Evidências apontam que o fortalecimento de sistemas locais de saúde, integrados em Redes de Atenção à Saúde (RAS), oferece potencialidades para a integração dos serviços e foco na qualidade do cuidado^(2-3)^. Os estudos destacam, ainda, que a integralidade, a partir das dimensões vertical e horizontal, garante: o acesso dos usuários aos diversos pontos da rede; articula a gestão da clínica individual e coletiva; fortalece os mecanismos de referência e contrarreferência; melhora a eficiência e a racionalidade dos serviços; além de gerar economia de escala e de escopo, contribuindo para o acesso e a sustentabilidade do sistema de saúde.

Os autores consideram que a articulação, coordenação e regulação em rede remetem à noção da interdependência dos profissionais e serviços de atenção à saúde, constituindo-se em um instrumento de equilíbrio entre a oferta e a demanda, promovendo uma melhor efetividade dos indicadores de saúde. Ressaltam, ainda, a importância da intersetorialidade do sistema de proteção social para as populações que vivem em condições de vida e de saúde desfavoráveis, que necessitam de políticas de transferência de renda para sobreviverem e manterem-se em tratamento de saúde^(2-3)^, a exemplo de quem adoece por tuberculose.

Dessa forma, a implementação das diretrizes organizacionais da descentralização e da regionalização, a partir dos anos 90, proporcionou novos avanços e desafios na estruturação organizacional do SUS^(4)^. Nesse arranjo organizacional, toma-se a APS como centro de comunicação, que pode colaborar para a interação da prática do cuidado individual e coletivo na perspectiva da vigilância em saúde, em território único, a partir das articulações institucionais baseadas nas potencialidades locais^(1)^.

Assim, a regionalização adquire visibilidade no espaço público, por tensionar as pautas políticas ao induzir os entes federados a assumirem o planejamento, gestão e financiamento das responsabilidades sanitárias. Com vistas à organização de um sistema descentralizado e regionalizado, focando nas respostas às condições de saúde da população, baseado no território e no fluxo assistencial dos serviços para conformação das RAS^(5)^.

Esse processo político introduziu novos sentidos e formas de ocupação dos espaços de governança, ancorados em evidências científicas que apontam a necessidade de mudança dessas práticas. Nesse aspecto, a organização de programas verticais, centrados na fragmentação dos serviços e em ações universalizantes, dificulta o fortalecimento de espaços colaborativos e o deslocamento do eixo organizacional do SUS para o estabelecimento do planejamento integrado com ênfase na pluralidade^(4-10)^, fato percebido no programa de atenção à TB.

Assim, o modelo estruturante para o controle da TB é concebido por meio de ações que se conectam do âmbito nacional ao local, incorporados ao controle como unidade padrão para identificar, atender e vincular os adoecidos à APS^(6,8-9)^. Essa modelização do sistema de saúde, constituído por diversos pontos de atenção, avança, desde 2010, com o intuito de responder às necessidades de cuidado longitudinal e garantir serviços mediados pelas RAS às doenças crônicas transmissíveis e não transmissíveis^(2,4,8-9)^.

Dessa maneira, é nesse contexto de reorganização das RAS que a TB, enquanto doença transmissível, encontra descompasso entre o que é preconizado pelo Programa Nacional de Controle da Tuberculose (PNCT) e as ações desenvolvidas para o acompanhamento e cuidado na APS.

Dessarte, justifica-se a relevância da realização desse estudo ante a necessidade de avanços na implementação de um modelo organizacional que abrigue a pluralidade, nos espaços de governança, exercida por diferentes atores que compartilham suas diferenças e mostram suas singularidades, possibilitando o aparecimento do novo na atenção à TB no SUS. A introdução desse debate na agenda da saúde estabelece acolhida aos princípios e diretrizes da descentralização e regionalização, conferindo estabilidade positiva para sua incorporação pelos gestores, profissionais de saúde e usuários^(5,7)^.

Dessa forma, emerge o questionamento: como a APS interage com a estrutura verticalizada do PNCT, a partir da pluralidade dos arranjos locais, assegurando mudanças nos modos de cuidado?

O objetivo do estudo foi analisar a pluralidade do cuidado de pessoas adoecidas por TB na APS, no município do Rio de Janeiro.

## Método

### Tipo de estudo

Estudo qualitativo, apoiado na Análise de Discurso (AD) de filiação francesa^(11)^.

### Local

Pesquisa realizada na Coordenadoria Geral de Atenção Primária (CAP) 3.1, uma das dez CAPs da Secretaria Municipal de Saúde (SMS) do Rio de Janeiro (RJ). A população é de 886.551 pessoas, distribuídas em 28 bairros, em que 50% desses apresentam alta densidade demográfica, acima de 12.517, e contêm 32 Unidades de Atenção Primária (UAPs).

### Período

A coleta de dados ocorreu em dois períodos distintos: segundo semestre de 2017 e primeiro semestre de 2021. Esse tempo estendido advém de inúmeras intercorrências, dada a disponibilidade de agenda dos entrevistados e a pandemia de Covid-19.

### Critérios de seleção

Os participantes foram selecionados intencionalmente, obedecendo aos critérios: gestores envolvidos com a implementação da APS, na SMS e na CAP 3.1; gestores responsáveis pela linha de cuidado da tuberculose, na CAP 3.1; profissionais (médicos e enfermeiros) com experiência no acompanhamento de pessoas adoecidas e em tratamento de TB, com alta, por cura ou abandono, nos territórios das UAPs.

### Participantes

Foram selecionados 15 participantes: oito homens e sete mulheres que se declararam cisgênero, sendo dois gestores, quatro médicos, quatro enfermeiros e cinco usuários, com idades entre 18 e 65 anos. Todos foram codificados a partir da posição sujeito ocupado, garantindo seu anonimato. Na seleção, contou-se com o apoio da CAP, auxiliando na identificação dos indivíduos que atendiam aos critérios de inclusão do estudo, os quais foram contatados por *e-mail* e *WhatsApp*. Não houve recusas para a participação.

### Instrumento de coleta de dados

Utilizaram-se dois roteiros semiestruturados, com perguntas voltadas a gestores, profissionais de saúde e usuários com TB, elaborados pelos pesquisadores. Os roteiros abordavam a compreensão da política de atenção à TB e sua implementação; a comunicação e vínculo entre profissionais e usuários, além da experiência de autocuidado dos usuários. As entrevistas, gravadas com duração de 20 a 60 minutos, ocorreram em UAPs e SMS, definidos pelos entrevistados. Não foi necessário retomar entrevistas.

### Tratamento e análise dos dados

Na organização do material empírico utilizou-se o *softwareATLAS.ti 8*, com a criação de um único projeto denominado de Unidade Hermenêutica (UH), constituído pelas entrevistas, que corresponderam aos documentos primários. Para a análise utilizou-se o dispositivo da AD, seguidos dos três níveis. Na primeira etapa aconteceu a passagem da superfície linguística para o objeto discursivo, ocorrendo a dessuperficialização do texto que considera a materialidade linguística: o como se diz e o quem diz e em que condições de produção. Na segunda etapa, houve a passagem do objeto discursivo para o processo discursivo: busca-se compreender os sentidos produzidos pela primeira etapa e o delineamento das formações discursivas, a partir de uma posição e conjuntura socio-histórica, que determina o que pode e deve ser dito. Por fim, na terceira etapa ocorreu o delineamento da formação discursiva e sua relação com a ideologia: acontece concomitante à segunda etapa, relaciona-se às formações discursivas distintas da formação ideológica, a qual é um conjunto complexo de atitudes e de representações relacionadas direta ou indiretamente às posições de classe em conflito, umas em relação às outras^(11)^.

As análises foram realizadas de forma colaborativa por quatro pesquisadores, com expertise em estudos qualitativos e no campo da saúde coletiva. A experiência acadêmica e profissional desses pesquisadores possibilitou uma compreensão detalhada das ações do PNCT, tanto antes quanto após a sua descentralização para a APS carioca. A codificação dos dados e a formulação dos enunciados aconteceu de maneira interativa, permitindo que as múltiplas perspectivas e interpretações fossem discutidas e acordadas coletivamente. Já a integração das reflexões de cada pesquisador, bem como as anotações de campo, refletiu-se na complexidade dos discursos sobre o cuidado da pessoa com tuberculose.

A AD, enquanto referencial teórico-metodológico, tem em vista colocar nos entremeios textuais uma leitura que libera os sentidos do discurso, por meio da sua materialidade, no encontro do histórico com o linguístico^(12-16)^; compreende o discurso como um objeto simbólico que se move nas falas de sujeitos, produzindo sentidos que possibilitam a permanência, a continuidade, o deslocamento e a transformação tanto do homem quanto de sua realidade^(17-19)^.

Na AD, o dispositivo teórico de análise segue a construção de um *corpus* que visa mostrar como um discurso funciona na produção de sentidos^(11,20)^. Os recortes discursivos são fragmentos da situação discursiva, dada pelas condições de produção nos contextos imediato e amplo e pelas interlocuções contextuais que envolvem sujeito, situação e memória^(20-23)^.

Assim, é na cadeia de acontecimentos históricos e de processos de significação que os discursos são produzidos^(11,17,24)^, tanto ela cronologia dos fatos históricos como pelas condições de produção, que permitem reconhecer a historicidade no texto^(13,21-22)^. Nesse contexto, as condições de produção caracterizam o processo discursivo, visibilizando tanto a paráfrase quanto a polissemia como constitutiva das Formações Discursivas (FDs). É do caráter heterogêneo e aberto das posições-sujeito que emerge a possibilidade de reorganização e de movimentação de saberes^(25-27)^.

As contribuições dos saberes das FDs para esse estudo estão nos constantes deslocamentos de sentidos que apontam o pré-construído e exterior ao sujeito discursivo, definindo aquilo que vem de outro lugar, sem que ele tenha consciência desse processo. Esse já dito, que fala antes, em outro lugar, através da memória discursiva, é o interdiscurso. É por meio da imbricação do já dito e o discurso em enunciação (intradiscurso) que se constitui o dizível^(13,17,19,23)^.

Desse modo, foram utilizados os conceitos de interdiscurso, condições de produção, memória, ideologia, e os processos parafrásticos e polissêmicos que movimentam continuamente a significação entre repetição e diferença, tornando presente a historicidade da linguagem, a qual é o acontecimento do texto como discurso^(21,23,25-26)^. Os recortes discursivos permitiram a mobilização dos conceitos e a interlocução com o referencial teórico-metodológico, que resultou na produção de sentidos, construção do dispositivo de interpretação e a formação dos três blocos discursivos, por meio das Sequências Discursivas (SDs)^(11,20)^.

Para ancorar as FDs das condições de produção mais amplas^(28)^, no campo da política pública da saúde, fez-se um diálogo com o referencial teórico de Hannah Arendt, para quem o espaço público é o lugar das discussões, tomadas de posição e opiniões, onde a relação entre os homens acontece^(7)^. Nesta ação os indivíduos se tornam atores, interagem mutuamente e comunicam aos outros quem são^(29)^.

Em relação à centralidade da política arendtiana, esta está na própria ação que acontece quando os indivíduos agem em conjunto, aberta à espontaneidade na busca pela distinção da singularização, marcada pela pluralidade^(7,29-30)^. Assim, nasce o conceito de natalidade, que pode ser compreendido como o ato ou efeito de instaurar novos começos, sob o pressuposto de que todos os indivíduos são novos começos por si sós, por chegarem pelo nascimento em um mundo que os precede e que será transformado por eles. Por isso, também lhes é facultado, pela ação, introduzir algo novo no mundo^(7,30-31)^.

Ressalta-se que a dimensão de pluralidade, espaço público e natalidade^(7,30-32)^ tem relevância, nesse contexto, porque permite enxergar que a ação compreende um começo que pode resultar na introdução de algo novo no mundo que, por ser incontrolável e imprevisível, não se pode determinar seu curso e nem prever seu desfecho. Por isso, essa linha de pensamento lança luz sobre questões silenciadas, controvérsias, opacidade e tensões que se articulam, movimentando os sentidos dos discursos circulantes nas FDs^(33-35)^.

### Aspectos éticos

A pesquisa foi aprovada pelos Comitês de Ética da SMS/RJ e da Universidade Federal do Rio de Janeiro-UFRJ (parecer nº 4.332.298). Todos os participantes assinaram o Termo de Consentimento Livre e Esclarecido (TCLE), e seus dados foram anonimizados por meio de codificação.

## Resultados

O primeiro enunciado explora como as memórias discursivas dos gestores influenciam a repetição e a estrutura verticalizada dos serviços e ações no tratamento da TB, destacando como as condições de produção dos discursos dos gestores interferem na forma como são organizados os serviços e ações para o tratamento da tuberculose (TB).

Ao analisar os discursos que circulam entre os gestores, identificaram-se marcas linguísticas que revelam contradições e desafios. Essas marcas podem ser observadas a seguir: *Meio policialesco, digamos assim… Quase, né? E pelo menos, assim, era a minha opinião. Continua sendo... Não acho que seja uma questão o Programa da Tuberculose ser normativo, entendeu?* (G1). Este discurso reflete a naturalização das práticas históricas e normativas PNCT, ancoradas em documentos oficiais do Ministério da Saúde.

A APS, nesse contexto, assume um papel central como ordenadora do cuidado, promovendo uma abordagem mais próxima do usuário, com maior foco na continuidade e na integralidade do cuidado, como pode ser observada em outra posição, no caso, gestor: *Então. Eu acho que, assim: bastante coisa conseguiu avançar, né? principalmente se a gente for pensar em quanto era restrito o acesso desse usuário, de se deslocar de lugar distante, sem dinheiro de passagem, pra vir numa Unidade secundária, agendar uma consulta… E a Equipe de Saúde da Família, ela tá dentro, né? praticamente dentro da casa do Usuário, né? O usuário praticamente mora dentro da Unidade de Saúde da Família porque a ligação é muito próxima* (G2). Este discurso mostra a ressignificação da política pública, destacando avanços no acesso ao tratamento por meio da proximidade e acessibilidade proporcionadas pela APS.

O segundo enunciado faz refletir sobre como a memória discursiva, associada à posição do sujeito usuário, pode moldar diferentes trajetórias na produção da vida, no contexto da APS. No caso dos usuários do sistema de saúde, essa memória abrange suas experiências, narrativas e percepções acumuladas ao longo do tempo. Ela influencia como os indivíduos compreendem e interagem com o sistema de saúde, formando suas expectativas e ações.

Isso é particularmente evidente no discurso da posição sujeito usuário, que compartilha sua experiência com o tratamento da tuberculose: *Suspender a gente não vai, porque a gente não pode parar o tratamento... É mais uma bomba, mas, assim: hoje eu consigo superar a cada… tranco que a vida me dá, porque eu tenho profissionais que me orientam muito. Hoje entendo um pouco o que que é a Tuberculose. É difícil lidar com ela…Então imagina: um pagode… um baile… besteira… cerveja. Você parar isso tudo da sua vida. Você parar de beber, É difícil! …quando eu comecei o meu tratamento era começo de novembro. Início de verão. Festa. Final de ano. Então foi… eu tive muito atrito familiar com isso* (U4). O discurso desvela a resistência a práticas burocráticas e despersonalizadas, sublinhando a necessidade de um cuidado adaptado às realidades dos usuários.

Na SD a seguir, percebe-se como essa memória discursiva se entrelaça com as experiências e desafios cotidianos, revelando a pluralidade e a capacidade dos indivíduos manifestarem-se nos espaços públicos a partir de suas vivências e existências, apontando a interseção entre as necessidades pessoais e as políticas públicas. Ressalta como as experiências individuais estão conectadas a contextos mais amplos de políticas de saúde: *Tudo envolve o governo, né? Tudo envolve a política, né? E o que acontece? …Então eu acho que isso aí a pessoa precisa valorizar mais. Ela tem que segurar essa oportunidade porque a morte, ela não é brincadeira. Morreu, acabou. Entendeu? Enquanto ela tá viva, ela tá tendo oportunidade de se tratar, de se cuidar…Surge certas situações… como é o meu caso… Eu tenho quatro filho, né? Às vezes, como foi o meu caso, a minha casa pegou fogo… Hoje… Eu tô há 15 dias sem tomar esse remédio. Entendeu? Então de lá pra cá aconteceu um fato de eu passar a madrugada toda com febre ontem. Minha filha ontem também passou mal… Entendeu?* (U2).

O discurso de U1 revela a memória discursiva do usuário do SUS, em que a obediência às instruções médicas é uma constante: *Eu faço o que ela manda! Eu chego lá: “Ó, toma o remédio aí!” E tal… Eu vejo o dia que eu comecei, né? o dia 15 de junho… Mas ela fala: “Ó, semana que vem…” Acho que hoje eu vou trocar de remédio. Eu vou pegar mais… É. Eu vou pegar um outro remédio que tem duas medicações, se eu não me engano. Então… o que ela falar eu faço!* (U1). Evidencia-se um padrão de comportamento regular no contexto do programa de controle da TB.

O discurso de U5 destaca os efeitos de sentidos na trajetória de tratamento da TB, revelando tanto as dificuldades enfrentadas pelo usuário, ao lidar com uma doença crônica, quanto o papel fundamental da APS em seu processo de recuperação: *Olhe, realmente tô sendo muito bem tratado aqui. Medicamento toda semana eu pego. Melhorei 100%, porque eu cheguei aqui com 43 quilo. Tô com 53 e… 400! Pesei hoje. Olha, isso aí foi uma… um descuido meu. Eu já tive a primeira vez e eu…num engano que teve o ano passado, eu achei que a enfermeira tinha me dado alta. Só que ela não tinha me dado alta ainda do medicamento. Aí eu abandonei o tratamento!* (U5).

O discurso de U3 destaca a complexidade do tratamento de doenças crônicas, enfatizando que a recuperação não se resume à prescrição e administração de medicamentos: *É, porque às vezes… Olha só: às vezes, tem muita gente que não se cura porque não tem ninguém que ajude aquela pessoa… Pensa que é só chegar aqui, o médico passar o remédio, tomou… Não. Então se a pessoa der força, a pessoa continua…*(U3).

O terceiro enunciado destaca como as memórias dos profissionais de saúde são acionadas para criar eventos discursivos significativos. Essas memórias discursivas envolvem a repetição e o deslocamento de experiências, caracterizadas por desvios, falhas, equívocos e contradições. Esses elementos conferem sentido às experiências vivenciadas pelos profissionais de saúde ao atuarem em um espaço público, cujo objetivo é organizar serviços e inovar práticas sanitárias para enfrentar condições crônicas.

A partir dessas condições de produção, é possível refletir como as memórias discursivas influenciam a forma como os médicos percebem e enfrentam os desafios em suas práticas cotidianas no contexto da APS: *Então eu acho que nesse sentido, né?, considerando que a gente não tá nas condições adequadas pra Atenção Primária, acho que o PNCT vai ficando fora da realidade. [Ri.] Vai ficando inadequado pra realidade atual da Atenção Primária. E isso pensando no Rio de Janeiro, que tem investimento, né? (M4).* Esse discurso destaca a desconexão entre o PNCT e a realidade da APS, no Rio de Janeiro, onde há investimentos, mas ainda assim as condições não são ideais.

Esta SD apresenta a importância de ajustar o tratamento da tuberculose, considerando a temporalidade e o ritmo de transmissão do bacilo, bem como a rede de suporte social do usuário: *É, por conta da transmissão, né? Então a gente tem que garantir que ele tome esses primeiros 15 dias, pra ele não transmitir mais, né? E, dependendo, do caso, a gente avalia, né? Às vezes a gente faz liberação no segundo mês, no terceiro mês… semanal… Se for uma pessoa que tem uma rede de apoio boa, uma família, né?* (E3)*.* Esse discurso reflete como as memórias e experiências dos enfermeiros orientam suas práticas e decisões, moldando a forma como enfrentam desafios no tratamento de condições crônicas como a tuberculose.

Nesta próxima análise, o discurso do médico aborda a fragmentação do cuidado e a tomada de decisões baseadas no histórico do usuário, que vai além de sua presença nos serviços de saúde: *Ele começou a tratar no presídio, veio pra uma equipe, tratou mais ou menos, aí terminou o tratamento comigo de uma forma também que ele não ’tava tomando a medicação correta, mas acabamos dando alta porque o escarro dele era negativo, ele tinha mais ou menos completado o período de tratamento… A gente deu alta. Menos de quatro meses depois ele recidivou, voltou, e aí, né? o início do tratamento muito bem, e a partir de dois meses, mais ou menos, começou a ter os mesmos problemas...*(M3). Destaca-se como o conhecimento do percurso pregresso do usuário influencia as decisões médicas, mostrando as dificuldades em se manter a continuidade e a eficácia do tratamento em contextos fragmentados.

Outro êxito foi compreender como o processo de descentralização das ações da tuberculose para as UAPs provoca novas interpretações e abordagens no cuidado, influenciadas tanto pelos discursos dos profissionais quanto pelo contexto específico em que atuam: *sou responsável pela linha de cuidado tem um ano e dois meses... Trabalhar a política é uma coisa complicada, e, com o usuário, pela fala do que é ser política pra eles… Então, quando eu cheguei na estratégia para começar essa linha de plano de cuidado de TB, tentei primeiro ver o território* (E2).

Nessa última SD, tem-se o reconhecimento das singularidades no contexto da APS. O discurso do médico ao acolher essas singularidades e operar com afeto, pode liderar o processo de cuidado, provocando rupturas nas práticas tradicionais e possibilitando abordagens efetivas, mediadas pela pluralidade: *Eu acho que a gente rastreia pouco. Mas os pacientes que são rastreados e têm diagnóstico positivo, acho que a partir daqui, da Atenção Primária, ela faz toda a diferença no cuidado, porque é um tratamento longo, que precisa de vínculo... Precisa de afeto pra entender quais são as limitações do cuidado… Entender as peculiaridades de cada paciente, de cada família… É… Acho que nesse sentido a Atenção Primária pode dar as cartas, entendeu?* (M2).

## Discussão

O estudo analisou as incongruências entre as normas do PNCT e a necessidade de uma abordagem mais plural no cuidado de pessoas com tuberculose na APS, oferecendo subsídios para profissionais e gestores de saúde identificarem os diversos significados desse adoecimento na população, promovendo maior equidade na atenção à saúde.

No primeiro enunciado, observa-se que a gestão municipal prioriza a política centralizada do PNCT, articulando as UAPs como unidades executantes das ações fragmentadas por meio das equipes da Estratégia Saúde da Família (ESF)^(8-9)^.

Nas condições de produção dos discursos, as memórias discursivas dos gestores influenciam a organização dos serviços de saúde, trazendo a reflexão de que o Rio de Janeiro ainda possui uma APS seletiva que pode estar baseada em resquícios históricos^(4)^.

Destaca-se que as FDs dos gestores no SUS do Rio de Janeiro geraram diferentes interpretações, invisibilizando as tensões e diferenças vividas por profissionais e usuários em face do discurso homogêneo das práticas universalizantes. Segundo Arendt, o espaço público é essencial para a pluralidade, singularidade e distinção, e nesse sentido, o espaço público, na acepção da autora, é o espaço por excelência do viver plural, da singularidade e da distinção^(7)^.

O reconhecimento da APS como centro de comunicação das RAS e coordenadora do cuidado pode trazer pluralidade aos arranjos institucionais, melhorar o acesso, a equidade e a humanização da assistência. Isso contribui para o avanço das políticas públicas de saúde, especialmente no enfrentamento de doenças crônicas como a TB^(2,4)^.

Assim, os arranjos plurais e a discursividade dos gestores permitem questionar e problematizar a hegemonia do tratamento da TB, conectando-se com outros discursos no território onde a vida acontece^(23)^.

No segundo enunciado, os discursos analisados revelam uma variedade de significados em torno da experiência dos usuários no tratamento da tuberculose, destacando a heterogeneidade^(35)^ das suas vivências. A posição dos usuários é constantemente ressignificada pelos discursos circulantes, o que permite que cada pessoa vivencie o tratamento de forma única, muitas vezes em desacordo com o padrão normatizado pelas políticas de saúde. Esses discursos também refletem as diversas formas de resistência que surgem na adesão ao tratamento, mostrando que suas ações e pensamentos são moldados por interações.

Nesse sentido, estudos mostram que considerar as experiências vividas dos usuários com TB é essencial para o sucesso do tratamento, fortalecendo a relação entre usuário e equipe de saúde. Outro aspecto destacado em diferentes estudos é a importância de políticas de transferência de renda^(36-38)^ para garantir o desfecho satisfatório do tratamento.

Ante a representação da doença para o adoecido por TB, o suporte familiar, social e da equipe de saúde é fundamental^(39)^ para o tratamento adequado, embora os usuários possam sentir que seu espaço está sendo invadido^(37)^. Assim, estudos destacam a necessidade de os profissionais de saúde reavaliarem continuamente suas atividades e competências, para proporcionar um cuidado holístico e integral^(36-37)^, na perspectiva da atenção centrada no usuário.

Nessa perspectiva, a pluralidade permite romper os limites do controle e criar novas relações na APS, estabelecendo um espaço público com o potencial de instaurar o novo^(7,30)^. Assim, a comunicação oportuna nas redes de cuidado em saúde^(4)^ destaca o autocuidado apoiado, como sendo um processo crucial para a participação e corresponsabilidade do indivíduo pela sua saúde.

Entretanto, devido aos efeitos colaterais e ao impacto social da TB, usuários frequentemente experienciam sofrimento mental, como depressão, que afetam o tratamento^(40)^. Com tal acometimento, a falta de suporte social e familiar pode levar à interrupção do tratamento, podendo gerar inúmeros transtornos para o adoecido, sendo, portanto, imprescindível a educação permanente dos profissionais para cuidar desses usuários^(37)^.

No terceiro enunciado, observou-se que as redes de memórias dos profissionais geram acontecimentos^(23)^ que influenciam seus discursos sobre o cuidado em saúde, sugerindo a inclusão da pluralidade nas práticas. Esses discursos conectam-se aos conceitos de espaço público^(7)^ e natalidade^(30)^, revelando a capacidade de iniciar algo novo em meio à complexidade das interações humanas.

Nessa teia discursiva, os discursos dos profissionais revelam contradições e aberturas que permitem o reconhecimento do sujeito singular adoecido por TB no espaço público, fazendo circular novas memórias. Essa abertura pode ser vista pela APS como uma chave de diálogo vinculada aos acontecimentos cotidianos^(41)^. As condições de existência das pessoas adoecidas, que dão sentido à luta pela vida e à revisão da padronização do controle, podem gerar reflexão na capacidade dos profissionais acolherem as repercussões de uma doença crônica^(6,9)^, como a TB, cujo tratamento e cuidado exigem um longo período^(39)^.

Logo, a TB, sendo uma preocupação de saúde pública que exige um tratamento prolongado, não pode ser enfrentada apenas com políticas focalizadas no término deste. Afinal, ignorar os determinantes sociais pode levar ao fracasso do tratamento^(37-38,42)^.

Nesse sentido, nos cenários pesquisados, a APS carioca desafia as ações autoritárias do PNCT, que se baseiam no controle hierarquizado. Essas ações utilizam práticas universais, reconhecidas por gestores, profissionais e usuários como uma linguagem específica do programa que autoriza seu modo de operar. Esse enfoque atribui um valor social ao discurso sobre a TB, reforçando práticas centradas no controle do tratamento e limitando as relações^(13)^.

Ao longo deste estudo, foi possível compreender que existem diferentes matrizes discursivas nos discursos circulantes. As matrizes parafrásticas fecham o discurso em um controle unilateral, enquanto as matrizes polissêmicas abrem o discurso da APS para a autonomia por meio da pluralidade. A presença de pensamentos diferentes e plurais nos espaços públicos pode gerar novos discursos que expressam formas inclusivas e participativas de cuidado.

Neste estudo, o caminho para romper com o controle estruturante na APS foi encontrado no diálogo com as ideias de Arendt sobre espaço público, pluralidade e natalidade. Essas ideias emergem das formações discursivas dos usuários e profissionais^(30-31)^, promovendo um ambiente mais poroso e centrado nas singularidades dos sujeitos.

As implicações para o campo da saúde e das equipes de profissionais incluem a possibilidade de promover novos começos e um agir político que reconheça a TB como uma doença crônica. Esse reconhecimento facilita a criação de novas conexões e práticas baseadas nos atributos da APS, resgatando a possibilidade de se tornar saudável através da pluralidade.

Nessa direção, o estudo sobre o cuidado de usuários com tuberculose multidroga resistente (TB-MDR) destaca a importância de uma abordagem centrada no usuário, valorizando a pluralidade de experiências. A centralidade do sujeito no cuidado permite aos profissionais criar e manter vínculos significativos com os usuários, superando as limitações impostas por protocolos rígidos e normas padronizadas^(43)^.

Por fim, as limitações do estudo estão relacionadas à amostra, que foi selecionada por conveniência. Embora os dados coletados tenham permitido compreender a perspectiva dos adoecidos por TB, não foram coletadas informações de familiares e pessoas próximas, que constituem a rede de suporte social dos adoecidos por TB. Recomenda-se que futuros estudos incluam informações da rede de suporte social dos adoecidos por TB. Essa abordagem reflexiva visa melhorar a qualidade da pesquisa e a relevância dos achados para intervenções mais eficazes e inclusivas no cuidado da TB.

## Conclusão

Este estudo analisou como o controle da tuberculose (TB), caracterizado por um modelo hierarquizado e normativo, pode apagar a individualidade dos usuários, tornando invisíveis sentimentos como desamparo, angústia, isolamento e medo da morte. A abordagem do Programa Nacional de Controle da Tuberculose (PNCT), com sua ênfase em manuais, técnicas e uma linguagem universal, contrasta com os cuidados produzidos na Atenção Primária à Saúde (APS), que valoriza as singularidades das realidades locais e os territórios vivos onde a vida acontece.

Nas interpelações da APS ao PNCT, observa-se uma disputa constante entre uma política plural, que valoriza as diferenças e organiza as RAS, de maneira a garantir o direito democrático à saúde e o modelo normativo do PNCT. A APS, com seus atributos, visa reconhecer e atender as singularidades dos usuários, propondo um cuidado centrado nas necessidades individuais e nas especificidades de cada território.

Para fortalecer essa abordagem plural e democrática, é fundamental apoiar os espaços embrionários de governança, resultantes da descentralização e regionalização da saúde. Esses espaços devem promover o protagonismo dos atores locais, vinculando-se à pluralidade no cuidado centrado no usuário. Essa estratégia contribuirá para assegurar o direito democrático à saúde de maneira mais assertiva, atendendo às necessidades específicas dos usuários e promovendo um cuidado mais humano e eficaz.

# Translation: La atención primaria en contexto con la pluralidad en el cuidado de personas con tuberculosis

**DOI:** 10.1590/1518-8345.6978.4472

**Authors:** Edna Ferreira Santos, Fátima Teresinha Scarparo Cunha, Pedro Fredemir Palha, Afranio Lineu Kritski

**Affiliations:** 1 Universidade Federal do Rio de Janeiro, Faculdade de Medicina, Rio de Janeiro, RJ, Brasil; 2 Secretária Municipal de Saúde, Superintendência de Atenção Primária, Coordenação de Doenças Crônicas Não Transmissíveis, Rio de Janeiro, RJ, Brasil; 3 Universidade Federal do Estado do Rio de Janeiro, Escola de Enfermagem Alfredo Pinto, Rio de Janeiro, RJ, Brasil; 4 Universidade de São Paulo, Escola de Enfermagem de Ribeirão Preto, Centro Colaborador de la OPS/OMS para el Desarrollo de la Investigación en Enfermería, Ribeirão Preto, SP, Brasil

**Keywords:** Política Pública, Atención Primaria de Salud, Semántica, Tuberculosis, Servicios de Salud, Atención a la Salud

## Abstract

**Objetivo:**

analizar la pluralidad del cuidado de personas enfermas de tuberculosis en la Atención Primaria a la Salud, en el municipio de Rio de Janeiro.

**Método:**

se trata de un estudio cualitativo, realizado entre 2017 y 2021; se utilizó el Análisis de Discurso como referencial teórico-metodológico. Fueron recolectados datos de gestores, profesionales de la salud y usuarios por medio de entrevistas semiestructuradas, categorizadas con el *software* ATLAS.ti 8. Para dar base a las Formaciones Discursivas, en el campo de la salud, se dialogó con los aportes teóricos del espacio público, pluralidad **y** natalidad, discutidos por Hannah Arendt.

**Resultados:**

los discursos analizados revelan la complejidad y los desafíos en la organización y ejecución de las prácticas de cuidado a la persona enferma con tuberculosis, destacándose la importancia del contexto histórico, social e individual de gestores, usuarios y profesionales de la salud. Se identificaron desafíos en la operacionalización de las Redes de Atención a la Salud y en la búsqueda de la singularidad de los usuarios.

**Conclusión:**

en las interacciones entre la Atención Primaria a la Salud y el Programa Nacional de Control de Tuberculosis existe una constante disputa entre las políticas que valorizan la pluralidad, la organizan de las Redes de Atención a la Salud y las que consideran el derecho democrático, en los espacios públicos.

## Destacados

**(1)** cuidado de personas con tuberculosis debe ser considerado a partir de la pluralidad. **(2)** Cambios en el tratamiento universal favorecen el cuidado centrado en el usuario. **(3)** El cuidado para enfermedades crónicas es complejo y requiere organización en redes temáticas. **(4)** Sistemas universales organizados en redes favorecen prácticas innovadoras en el cuidado. **(5)** La participación y emancipación de los sujetos enfermos potencializa el cuidado de la salud.

## Introducción

El modelo de atención que incentiva la distribución e implementación del Sistema Único de Salud (SUS) es la Atención Primaria a la Salud (APS), por resultar en cambios en las políticas locales de gestión y organización de las prácticas de atención^(1)^. En ese sentido, el escenario sanitario actual exige cambios importantes, tanto para el manejo de las condiciones infecciosas y agudas como de las crónicas, por medio de estrategias eficaces, constituyéndose en un desafío para la perspectiva económica y la organización de la atención a la salud. Considerando el objetivo de obtener mejores indicadores sanitarios en los sistemas de salud^(2)^, especialmente, en el control de la Tuberculosis (TB) en la APS.

Evidencias apuntan que el fortalecimiento - de sistemas locales de salud, integrados en Redes de Atención a la Salud (RAS) - ofrece potencialidades para la integración de los servicios y enfoque en la calidad del cuidado^(2-3)^. Además, los estudios destacan que la integralidad, a partir de las dimensiones vertical y horizontal, garantizan: el acceso de los usuarios a los diversos puntos de la red; permiten articular la gestión de la clínica individual y colectiva; fortalece los mecanismos de referencia y contra-referencia; mejora la eficiencia y la racionalidad de los servicios; y además, genera economía de escala y de alcance, lo que contribuye para el acceso y la sustentabilidad del sistema de salud.

Los autores consideran que la articulación, coordinación y regulación de la red, se vinculan a la noción de la interdependencia de los profesionales y servicios de atención a la salud, constituyéndose en un instrumento de equilibrio entre la oferta y la demanda, promoviendo una mejor efectividad de los indicadores de salud. Además, destacan la importancia de la intersectorialidad del sistema de protección social, para las poblaciones que viven en condiciones de vida y de salud desfavorables, y que necesitan de políticas de transferencia de renta para que las personas puedan sobrevivir y mantenerse en tratamiento de salud^(2-3)^_,_ por ejemplo de personas con tuberculosis.

De esa forma, la implementación de las directrices organizacionales de descentralización y de regionalización, a partir de los años 90, proporcionó nuevos avances y desafíos en la estructuración organizacional del SUS^(4)^. En esa propuesta organizacional, se considera a la APS como centro de comunicación, que puede colaborar para la interacción de la práctica del cuidado individual y colectivo en la perspectiva de la vigilancia en salud, en territorio único, a partir de las articulaciones institucionales basadas en las potencialidades locales^(1)^.

Así, la regionalización adquiere visibilidad en el espacio público, por tensionar las pautas políticas al inducir a las instituciones federales a asumir la planificación, gestión y financiamiento de las responsabilidades sanitarias. Así, objetivándose una organización de un sistema descentralizado y regionalizado, enfocando en las respuestas a las condiciones de salud de la población, basado en el territorio y en el flujo asistencial de los servicios para la conformación de las RAS^(5)^.

Ese proceso político introdujo nuevos sentidos y formas de ocupación de los espacios de gobernanza, basados en evidencias científicas que apuntan la necesidad de realizar cambios en esas prácticas. En ese aspecto, verticalizar la organización de programas, centrados en la fragmentación de los servicios y en acciones universales, dificulta el fortalecimiento de espacios colaborativos y el dislocamiento del eje organizacional del SUS, para el establecimiento de una planificación integrada con énfasis en la pluralidad^(4-10)^, hecho percibido en el programa de atención a la TB.

Así, el modelo estructurante para el control de la TB es concebido por medio de acciones que conectan el ámbito nacional al local, incorporándolos al control como unidad estándar para identificar, atender y vincular los enfermos a la APS^(6,8-9)^. Ese modelo de sistema de salud, constituido por diversos puntos de atención, avanza, desde 2010, con el objetivo de responder a las necesidades de cuidado longitudinal y garantizar servicios mediados por las RAS a las enfermedades crónicas transmisibles y no transmisibles^(2,4,8-9)^.

De esa manera, es en ese contexto de reorganización de las RAS en que la TB, como enfermedad transmisible, está descompasada entre lo que es preconizado por el Programa Nacional de Control de la Tuberculosis (PNCT) y las acciones desarrolladas para el acompañamiento y cuidado en la APS.

De este modo, se justifica la relevancia de la realización de este estudio frente a la necesidad de avanzar en la implementación de un modelo organizacional que abrigue la pluralidad, en los espacios de gobernanza, ejercida por diferentes actores que comparten sus diferencias y muestran sus singularidades, posibilitando el aparecimiento de lo nuevo en la atención a la TB en el SUS. La introducción de este debate en la agenda de la salud establece permitirá acoger los principios y directrices de la descentralización y regionalización, dando estabilidad positiva para su incorporación por los gestores, profesionales de salud y usuarios^(5,7)^.

De esa forma, surge la pregunta: ¿Cómo la APS interactúa con la estructura vertical del PNCT, a partir de la pluralidad de los arreglos locales, asegurando cambios en los modos del cuidado?

El objetivo de este estudio fue analizar la pluralidad del cuidado a las personas enfermas de TB en la APS, en el municipio de Rio de Janeiro.

## Método

### Tipo de estudio

Estudio cualitativo, apoyado en el Análisis de Discurso (AD) de filiación francesa^(11)^.

### Local

Investigación realizada en la Coordinación General de Atención Primaria (CAP) 3.1, que es una de las diez CAPs de la Secretaría Municipal de Salud (SMS) de Rio de Janeiro (RJ). La población fue de 886.551 personas, distribuidas en 28 barrios, en que 50% de ellos presentan alta densidad demográfica, arriba de 12.517, y contienen 32 Unidades de Atención Primaria (UAPs).

### Período

La recolección de datos ocurrió en dos períodos distintos: en el segundo semestre de 2017 y primero semestre de 2021. En ese largo período ocurrieron numerosas intercurrencias, dada la disponibilidad de agenda de los entrevistados y el acontecimiento de la pandemia de Covid-19.

### Criterios de selección

Los participantes fueron seleccionados intencionalmente, obedeciendo a los siguientes criterios: gestores envueltos con la implementación de la APS, en la SMS y en la CAP 3.1; gestores responsables por la línea de cuidado de la tuberculosis, en la CAP 3.1; y, profesionales (médicos y enfermeros) con experiencia en el acompañamiento de personas enfermas y en tratamiento de TB, con alta, por cura o abandono, en los territorios de las UAPs.

### Participantes

Fueron seleccionados 15 participantes: 8 hombres y 7 mujeres que se declararon CIS. Los gestores fueron: cuatro médicos, cuatro enfermeros y cinco usuarios, con edades entre 18 y 65 años. Todos fueron codificados a partir de la posición sujeto ocupado, garantizando el anonimato. En la selección, se contó con el apoyo de la CAP, que auxilió la identificación de los individuos que atendían a los criterios de inclusión del estudio, los cuales fueron contactados por *e-mail* y *WhatsApp*, no hubo rechazos para la participación.

### Instrumento de recolección de datos

Se utilizaron dos guiones semiestructurados, conteniendo preguntas dirigidas a los gestores, profesionales de salud y usuarios con TB, elaborados por los investigadores. Los guiones abordaron la comprensión de la política de atención a la TB y su implementación; también la comunicación y vínculo entre profesionales y usuarios, además de la experiencia de autocuidado de los usuarios. Las entrevistas, fueron grabadas con duración de 20 a 60 minutos, las que ocurrieron en UAPs y SMS, definidos por los entrevistados. No fue necesario retomar las entrevistas.

### Tratamiento y análisis de los datos

En la organización del material empírico se utilizó el *software* ATLAS.ti-8, con la creación de un único proyecto denominado Unidad Hermenéutica (UH), constituido por las entrevistas que correspondieron a los documentos primarios. Para el análisis se utilizó el dispositivo de la AD, seguidos de los tres niveles. Primera etapa: pasaje de la superficie lingüística para el objeto discursivo, retirando la superficialidad del texto que lleva en consideración la materialidad lingüística (el cómo se dice, quién dice y en qué condiciones de producción). Segunda etapa: pasaje del objeto discursivo para el proceso discursivo, que considera los sentidos producidos por la primera etapa y el delineamiento de las formaciones discursivas, a partir de una posición y coyuntura socio-histórica, que determina lo que puede y debe ser dicho. Por último, la tercera etapa: delineamiento de la formación discursiva y su relación con la ideología, que ocurre concomitantemente con la segunda etapa y se relaciona con las formaciones discursivas distintas a la formación ideológica, que es un conjunto complejo de actitudes y de representaciones relacionadas directa o indirectamente a las posiciones de clase en conflicto, unas en relación a las otras^(11)^.

Los análisis fueron realizados de forma colaborativa por cuatro investigadores expertos en estudios cualitativos y en el campo de la salud colectiva. La experiencia académica y profesional de esos investigadores posibilitó una comprensión detallada de las acciones del PNCT, tanto antes como después de la descentralización de las APS cariocas. La codificación de los datos y la formulación de los enunciados acontecieron de manera interactiva, permitiendo que las múltiples perspectivas e interpretaciones fuesen discutidas y concordadas colectivamente. La integración de las reflexiones - de cada investigador, así como las anotaciones de campo - se reflejó en la complejidad de los discursos sobre el cuidado a la persona con tuberculosis.

La AD, como referencial teórico-metodológico, busca colocar en las entre líneas textuales una lectura que libera los sentidos del discurso, por medio de su materialidad, en el encuentro de lo histórico con lo lingüístico^(12-16)^; comprende el discurso como un objeto simbólico que se mueve en las declaraciones de sujetos, produciendo sentidos que posibilitan la permanencia, continuidad, desplazamiento y transformación, tanto del hombre como de su realidad^(17-19)^.

En la AD, al dispositivo teórico de análisis sigue la construcción de un *corpus* que objetiva mostrar cómo un discurso funciona en la producción de sentidos^(11,20)^. Los recortes discursivos son fragmentos de la situación discursiva, dado por las condiciones de producción en los contextos inmediato y amplio y por las interlocuciones contextuales que envuelven sujeto, situación y memoria^(20-23)^.

Así, es en la cadena de acontecimientos históricos y de procesos de significación que los discursos son producidos^(11,17,24)^, tanto por la cronología de los hechos históricos como por las condiciones de producción, que permiten reconocer la historicidad en el texto^(13,21-22)^. En ese contexto, las condiciones de producción caracterizan el proceso discursivo, visibilizando tanto la paráfrasis y la polisemia como constitutivas de las Formaciones Discursivas (FDs). Es del carácter heterogéneo y abierto de las posiciones-sujeto que emerge la posibilidad de reorganización y de movimiento de los saberes^(25-27)^.

Las contribuciones de los saberes de las FDs, para este estudio, están en los constantes desplazamientos de los sentidos que apuntan para lo pre-construido y lo exterior al sujeto discursivo, definiendo aquello que viene de otro lugar, sin que este tenga consciencia de ese proceso. Eso ya afirmado, que habla antes, en otro lugar, a través de la memoria discursiva, es el interdiscurso. Es por medio de la imbricación de lo ya dicho y el discurso en enunciación (intradiscurso) que se constituye lo decible^(13,17,19,23)^.

De ese modo, fueron utilizados los conceptos de interdiscurso, condiciones de producción, memoria, ideología, y procesos parafrásticos y polisémicos que mueven continuamente la significación entre repetición y diferencia, tornando presente la historicidad del lenguaje, que es el acontecimiento del texto como discurso^(21,23,25-26)^. Los recortes discursivos permitirán la movilización de los conceptos y la interlocución con el referencial teórico-metodológico, que resultó en la producción de sentidos, construcción del dispositivo de interpretación y la formación de los tres bloques discursivos, por medio de las Secuencias Discursivas (SDs)^(11,20)^.

Con la finalidad de dar base a las FDs de las condiciones de producción más amplias^(28)^, en el campo de la política pública de la salud, se hizo un diálogo con el referencial teórico de Hannah Arendt, para quien el espacio público es el lugar de las discusiones, tomadas de posición y opiniones, en donde la relación entre los hombres sucede^(7)^. En esta acción los individuos se tornan actores, interactúan unos con los otros y comunican a los otros quienes son^(29)^.

En relación a la centralidad de la política arendtiana, ella está en la propia acción que sucede cuando los individuos actúan en conjunto, abiertos a la espontaneidad, buscando la distinción de la singularización, marcada por la pluralidad^(7,29-30)^. Así, nace el concepto de natalidad que puede ser comprendido como el acto o efecto de instaurar nuevos comienzos, bajo la suposición de que todos los individuos son nuevos comienzos por sí solos, ya que llegan por el nacimiento en un mundo que los precede y que será transformado por ellos. Por eso también les es facultado, por la acción, a introducir algo nuevo en el mundo^(7,30-31)^.

Se destaca que la dimensión de pluralidad, espacio público y natalidad^(7,30-32)^ tienen relevancia, en ese contexto, porque permiten observar que la acción comprende un comienzo que puede resultar en la introducción de algo nuevo en el mundo que, por ser incontrolable e imprevisible, no se puede determinar su curso y ni prever su resultado. Por eso, esa línea de pensamiento lanza luz sobre cuestiones silenciadas, controversias, opacidad y tensiones que se articulan, moviendo los sentidos de los discursos circulantes en las FDs^(33-35)^.

### Aspectos éticos

La investigación fue aprobada por los Comités de Ética de la SMS/RJ y de la Universidad Federal de Rio de Janeiro-UFRJ (Parecer Nº: 4.332.298). Todos los participantes firmaron el Término de Consentimiento Libre e Informado (TCLI), y sus datos fueron anonimizados por medio de codificación.

## Resultados

El primer enunciado explora cómo las memorias discursivas de los gestores influencian la repetición y estructura vertical de los servicios y las acciones en el tratamiento de la TB, destacando cómo las condiciones de producción de los discursos de los gestores interfieren en la forma de organización los servicios y acciones para el tratamiento de la TB.

Al analizar los discursos que circulan entre los gestores, se identificaron marcas lingüísticas que revelan contradicciones y desafíos. Esas marcas pueden ser observadas a seguir: *Medio policial, digamos así… Casi, ¿No es verdad? Y por lo menos, así, era mi opinión. Continúa siendo... No creo que sea una cuestión que el Programa de la Tuberculosis sea normativo. ¿Entendió?* (G1). Este discurso refleja la naturalización de las prácticas históricas y normativas PNCT, basadas en documentos oficiales del Ministerio de la Salud.

La APS, en ese contexto, asume un papel central como ordenadora del cuidado, promoviendo un abordaje más próximo del usuario, con mayor enfoque en la continuidad y en la integralidad del cuidado, como puede ser observado en otra posición del gestor*: Entonces. Yo creo que, así: bastantes cosas conseguí avanzar. ¿No es verdad? principalmente si fuéramos a pensar en cuanto era restricto el acceso de ese usuario, para trasladarse de lugar distante, sin dinero del pasaje, para venir a una Unidad secundaria, agendar una consulta… Y el Equipo de Salud de la Familia, ella está adentro, ¿No es Verdad? prácticamente dentro de la casa del Usuario, ¿No es verdad? El usuario prácticamente vive dentro de la Unidad de Salud de la Familia porque el vínculo es muy próximo* (G2). Este discurso muestra la resignificación de la política pública, destacando los avances en el acceso al tratamiento por medio de la proximidad y accesibilidad proporcionadas por la APS.

El segundo enunciado permite reflexionar sobre como la memoria discursiva, asociada a la posición del sujeto usuario, puede moldar diferentes trayectorias en la producción de la vida, en el contexto de la APS. En el caso de los usuarios del sistema de salud, esa memoria abarca sus experiencias, narrativas y percepciones acumuladas a lo largo del tiempo. Ella influencia como los individuos comprenden e interactúan con el sistema de salud, formando sus expectativas y acciones.

Eso es particularmente evidente en el discurso de la posición sujeto usuario, que comparte su experiencia con el tratamiento de la tuberculosis: *No vamos a suspender, porque nosotras no podemos parar el tratamiento... Es más una bomba, pero, así: hoy consigo superarme a cada… tropiezo que la vida me da, porque tengo profesionales que me orientan mucho. Hoy entiendo un poco lo que es la Tuberculosis. Es difícil lidiar con ella…Entonces imagina: un fiesta… un baile… tontería… cerveza. Usted puede evitar eso de su vida. Parar de beber, es difícil! …cuando yo comencé mi tratamiento era el comienzo de noviembre. Inicio del verano. Fiesta. Final de año. Entonces fue… yo tuve mucha confusión familiar con eso.* (U4). El discurso revela la resistencia a prácticas burocráticas y despersonalizadas, subrayado la necesidad de un cuidado adaptado a las realidades de los usuarios.

En la próxima SD se percibe como esa memoria discursiva se entrelaza con las experiencias y desafíos cotidianos, revelando la pluralidad y capacidad de los individuos de manifestarse en los espacios públicos, a partir de sus vivencias y existenciales, apuntando la intersección entre las necesidades personales y las políticas públicas, resaltando como las experiencias individuales están conectadas a contextos más amplios de políticas de salud: *Todo envuelve al gobierno. ¿No es verdad? Todo envuelve la política. ¿No es verdad? ¿Y que sucede? …Entonces yo creo que eso ahí la persona precisa valorizar más. Ella tiene que asegurar esa oportunidad porque la muerte, no es un juego. Murió, acabó. ¿Entendió? En cuanto ella está viva, ella está teniendo oportunidad de tratarse, de cuidarse…Surgen ciertas situaciones… como es mi caso… Yo tengo cuatro hijos. A veces, como fue en mi caso, mi casa se incendio … Hoy… Yo estoy hace 15 días sin tomar ese remedio. ¿Entendió? Entonces de allá para acá sucedió el hecho de yo pasar la madrugada toda con fiebre, ayer. Mi hija ayer también pasó mal… ¿Entendió?* (U2).

El discurso de U1 revela la memoria discursiva del usuario del SUS, en el cual la obediencia a las instrucciones médicas es una constante: *Yo hago lo que ella manda. Yo llego allá: “Mira, toma el remedio ahí!” Y tal… Hoy, yo veo que comencé. ¿No es verdad? el día 15 de junio… Pero ella dice: “Mira, semana que viene…” Creo que hoy yo voy a cambiar de remedio. Yo voy a recibir más… Es. yo voy a recibir otro remedio que tiene dos medicaciones, si yo no me engaño. Entonces… lo que ella dice yo hago.* (U1). Se evidencia un patrón de comportamiento regular dentro del contexto del programa de control de la TB.

El discurso de U5 destaca los efectos de los sentidos en la trayectoria del tratamiento de la TB, revelando tanto las dificultades enfrentadas por el usuario, al lidiar con una enfermedad crónica, en lo que se refiere al papel fundamental de la APS, en su proceso de recuperación: *Mire, realmente estoy siendo muy bien tratado aquí. Medicamento toda semana yo recibo. Mejoré 100%, porque yo llegué aquí con 43 quilos. Estoy con 53 y… 400. Me pesé hoy. Mire, eso ahí fue una… un descuido mío. Yo ya tuve la primera vez y yo…en un engaño que tuve el año pasado, yo creí que la enfermera me había dado alta. Solo que ella no me había dado alta todavía del medicamento. Ahí yo abandoné el tratamiento.* (U5).

El discurso de U3 destaca a complejidad del tratamiento de enfermedades crónicas, enfatizando que la recuperación no se resume a la prescripción y administración de medicamentos: *Es, porque a veces… Vea solamente: a veces, hay mucha gente que no se cura porque no tiene a nadie que ayude a aquella persona… Piensa que es solo llegar aquí, el médico pasa el remedio, y lo toma… No. Entonces si la persona le da fuerzas, la persona continua…*(U3).

El tercero enunciado destaca como las memorias de los profesionales de la salud son accionadas para crear eventos discursivos significativos. Esas memorias discursivas contienen la repetición y el dislocamiento de experiencias, caracterizadas por desvíos, fallas, equívocos y contradicciones. Esos elementos le dan sentido a las experiencias experimentadas por los profesionales de salud al actuar en un espacio público, cuyo objetivo es organizar servicios e innovar prácticas sanitarias para enfrentar condiciones crónicas.

A partir de esas condiciones de producción, es posible reflexionar sobre cómo las memorias discursivas influencian la forma en que los médicos perciben y enfrentan los desafíos en sus prácticas cotidianas en el contexto de la APS: *Entonces yo creo que es en ese sentido, ¿No es verdad? considerando que nosotras no estamos en las condiciones adecuadas para la Atención Primaria, creo que el PNCT está quedando fuera de la realidad. [Ri.] Va quedando inadecuado para la realidad actual de la Atención Primaria. Y eso pensando en Rio de Janeiro, que tiene recursos. ¿No es verdad?* (M4). Ese discurso destaca la desconexión entre el PNCT y la realidad de la APS, en Rio de Janeiro, en donde hay recursos, pero a pesar de eso, las condiciones no son ideales.

Esta SD presenta la importancia de ajustar el tratamiento de la tuberculosis, considerando la temporalidad y el ritmo de transmisión del bacilo, así como la red de soporte social del usuario: *Si, por cuenta de la transmisión. ¿No es verdad? Entonces tenemos que garantizar que él tome esos primeros 15 días, para que él no transmita más. ¿No es verdad? Y, dependiendo, del caso, evaluamos. ¿No es verdad? A veces liberamos en el segundo mes, en el tercero mes… semanal… si es una persona que tiene una red de apoyo buena, una familia. ¿No es verdad?* (E3). Ese discurso refleja cómo las memorias y experiencias de los enfermeros orientan sus prácticas y decisiones, moldando la forma como enfrentan desafíos en el tratamiento de condiciones crónicas, como la tuberculosis.

En este próximo análisis, el discurso del médico aborda la fragmentación del cuidado y la toma de decisiones basadas en el histórico del usuario, que va más allá de su presencia en los servicios de salud: *Él comenzó a tratarse en el presidio, vino para un equipo, se trató más o menos, ahí terminó el tratamiento conmigo de una forma también que él no ’estaba tomando la medicación correcta, pero acabamos dando alta porque el esputo de él era negativo, él tenía más o menos completado el período de tratamiento… Nosotras le dimos alta. Menos de cuatro meses después tuvo la recidiva, volvió, y ahí. ¿No es verdad? El inicio del tratamiento muy bien, y a partir de dos meses, más o menos, comenzó a tener los mismos problemas...*(M3). Se destaca cómo el conocimiento del recorrido anterior del usuario influencia las decisiones médicas, mostrando las dificultades en mantener la continuidad y eficacia del tratamiento en contextos fragmentados.

Otro resultado positivo fue comprender cómo el proceso de descentralización de las acciones de la tuberculosis para las UAPs provoca nuevas interpretaciones y abordajes en el cuidado, influenciados tanto por los discursos de los profesionales como por el contexto específico en que actúan: *soy responsable por la línea de cuidado hace un año y dos meses... Trabajar la política es una cosa complicada, y, con el usuario, por la comprensión de lo que es la política para ellos… Entonces, cuando yo llegué en la estrategia para comenzar esa línea del plan de cuidado de TB, traté primero de ver el territorio* (E2).

En esta última SD, se reconocen las singularidades en el contexto de la APS. El discurso del médico al acoger esas singularidades y operar con afecto, puede liderar el proceso de cuidado, provocando rupturas en las prácticas tradicionales y posibilitando abordajes efectivos, mediadas por la pluralidad: *Yo creo que rastreamos poco. Pero los pacientes que son rastreados y tienen diagnóstico positivo, creo que a partir de aquí, de la Atención Primaria, ella hace toda la diferencia en el cuidado, porque es un tratamiento largo, que necesita de vínculo... Necesita de afecto para entender cuáles son las limitaciones del cuidado… Entender las peculiaridades de cada paciente, de cada familia… Es… Creo que en ese sentido la Atención Primaria puede dar las cartas. ¿Entendió?* (M2).

## Discusión

El estudio analizó las incongruencias entre las normas del PNCT y la necesidad de un abordaje más plural en el cuidado de personas con tuberculosis en la APS, ofreciendo subsidios para profesionales y gestores de salud para que puedan identificar los diversos significados de esa enfermedad en la población, promoviendo mayor equidad en la atención a la salud.

En el primero enunciado, se observa que la gestión municipal prioriza la política centralizada del PNCT, articulando las UAPs como unidades ejecutoras de las acciones fragmentadas, por medio de los equipos de la Estrategia Salud de la Familia (ESF)^(8-9)^.

En las condiciones de producción de los discursos, las memorias discursivas de los gestores influencian la organización de los servicios de salud, provocando la reflexión de que Rio de Janeiro todavía posee una APS selectiva que puede estar basada en el pasado histórico^(4)^.

Se destaca que las FDs de los gestores en el SUS de Rio de Janeiro generaron diferentes interpretaciones, provocando la invisibilidad de las tensiones y diferencias vividas por profesionales y usuarios frente al discurso homogéneo de las prácticas universales. Según Arendt, el espacio público es esencial para la pluralidad, singularidad y distinción y, en ese sentido, el espacio público, en la acepción de la autora, es el espacio por excelencia del vivir plural, de la singularidad y de la distinción^(7)^.

El reconocimiento de la APS como centro de comunicación de las RAS y coordinadora del cuidado puede traer pluralidad a los arreglos institucionales, mejorar el acceso, la equidad y la humanización de la asistencia. Eso contribuye para el avance de las políticas públicas de salud, especialmente en el enfrentamiento de enfermedades crónicas como la TB^(2,4)^.

Así, los arreglos plurales y el discurso de los gestores permiten cuestionar y problematizar la hegemonía del tratamiento de la TB, conectándose con otros discursos en el territorio en donde la vida sucede^(23)^.

En el segundo enunciado, los discursos analizados revelan una variedad de significados en torno de la experiencia de los usuarios en el tratamiento de la tuberculosis, destacándose la heterogeneidad^(35)^ de sus vivencias. La posición de los usuarios es constantemente dotada de nuevo significado por los discursos circulantes, lo que permite que cada persona experimente el tratamiento de forma única, muchas veces en desacuerdo con el estándar normal por las políticas de salud. Esos discursos también reflejan las diversas formas de resistencia que surgen en la adhesión al tratamiento, mostrando que sus acciones y pensamientos son moldados por interacciones.

En ese sentido, estudios muestran que considerar las experiencias vividas de los usuarios con TB es esencial para el éxito del tratamiento, que fortaleciendo la relación entre usuario y equipo de salud. Otro aspecto, destacado en diferentes estudios, es la importancia de políticas de transferencia de renta^(36-38)^ para garantizar el resultado satisfactorio del tratamiento.

Frente a la representación de la enfermedad para el enfermo de TB, el soporte familiar, social y del equipo de salud es fundamental^(39)^ para el tratamiento adecuado, a pesar de que los usuarios podrían sentir que su espacio está siendo invadido^(37)^. Así, estudios destacan la necesidad de los profesionales de salud de reevaluar continuamente sus actividades y competencias, para proporcionar un cuidado holístico e integral^(36-37)^, en la perspectiva de obtener una atención centrada en el usuario.

En esa perspectiva, la pluralidad permite romper los límites del control y crear nuevas relaciones en la APS, estableciendo un espacio público con el potencial de instaurar lo nuevo^(7,30)^. Así, la comunicación oportuna en las redes de cuidado de la salud^(4)^ destaca el autocuidado apoyado, como siendo un proceso crucial para la participación y corresponsabilidad del individuo por su salud.

Entre tanto, debido a los efectos colaterales y al impacto social de la TB, los usuarios frecuentemente experimentan sufrimiento mental, como la depresión que afectan el tratamiento^(40)^. Con ese acontecimiento, la falta de soporte social y familiar pueden llevar a la interrupción del tratamiento, lo que puede generar numerosos trastornos para el enfermo; por tanto, es imprescindible la educación permanente de los profesionales sobre el cuidado de esos usuarios^(37)^.

En el tercer enunciado, se observó que las redes de memorias de los profesionales generan acontecimientos^(23)^ que influenciaron sus discursos sobre el cuidado en salud, sugiriendo la inclusión de la pluralidad en las prácticas. Esos discursos se conectan a los conceptos de espacio público^(7)^ y natalidad^(30)^, revelando la capacidad de iniciar algo nuevo en medio a la complejidad de las interacciones humanas.

En esta red discursiva, los discursos de los profesionales revelan contradicciones y aberturas que permiten el reconocimiento del sujeto singular enfermo de TB, en el espacio público, permitiendo circular nuevas memorias. Esa abertura puede ser vista por la APS como una llave de diálogo vinculada a los acontecimientos cotidianos^(41)^. Las condiciones de existencia de las personas enfermas, que dan sentido a la lucha por la vida y a la revisión de la estandarización del control, pueden generar reflexiones sobre la capacidad de los profesionales de poder percibir las repercusiones de una enfermedad crónica^(6,9)^, como la TB, cuyo tratamiento y cuidado exigen un largo período^(39)^.

Luego, la TB - siendo una preocupación de salud pública que exige un tratamiento prolongado - no puede ser enfrentada apenas con políticas enfocadas en el término de la enfermedad. Pues, ignorar los determinantes sociales puede llevar al fracaso del tratamiento^(37-38,42)^.

En ese sentido, en los escenarios investigados, la APS carioca desafía las acciones autoritarias del PNCT, que se basan en el control jerarquizado. Esas acciones utilizan prácticas universales, reconocidas por gestores, profesionales y usuarios como un lenguaje específico del programa que autoriza su modo de operar. Ese enfoque atribuye un valor social al discurso sobre la TB, reforzando prácticas centradas en el control del tratamiento y limitando las relaciones^(13)^.

A lo largo de este estudio, fue posible comprender que existen diferentes matrices discursivas en los discursos circulantes. Las matrices parafrásticas cierran el discurso en un control unilateral; por otro lado, las matrices polisémicas abren el discurso de la APS para la autonomía por medio de la pluralidad. La presencia de pensamientos diferentes y plurales en los espacios públicos puede generar nuevos discursos que expresen formas inclusivas y participativas del cuidado.

En este estudio, el camino para romper el control estructurante de la APS, fue encontrado en el diálogo con las ideas de Arendt sobre el espacio público, la pluralidad y natalidad. Esas ideas surgen de las formaciones discursivas (FDs) de los usuarios y profesionales^(30-31)^, promoviendo un ambiente más permeable y centrado en las singularidades de los sujetos.

Las implicaciones para el campo de la salud y de los equipos de profesionales incluyen la posibilidad de promover nuevos comienzos y un actuar político que reconozca la TB como una enfermedad crónica. Ese reconocimiento facilita la creación de nuevas conexiones y prácticas basadas en los atributos de la APS, rescatando la posibilidad de tornarse saludable a través de la pluralidad.

En esa dirección, el estudio sobre el cuidado de usuarios con multidrogorresistentes (TB-MDR) destaca la importancia de un abordaje centrado en el usuario, valorizando la pluralidad de experiencias. La centralidad del sujeto en el cuidado permite a los profesionales crear y mantener vínculos significativos con los usuarios, superando las limitaciones impuestas por protocolos rígidos y normas estandarizadas^(43)^.

Por último, las limitaciones del estudio están relacionadas a la muestra, que fue seleccionada por conveniencia. A pesar de que los datos recolectados hubiesen permitido comprender la perspectiva de los enfermos por TB, no fueron recolectadas informaciones de familiares y personas próximas, que constituyen la red de soporte social de los enfermos de TB. Se recomienda que futuros estudios incluyan informaciones de la red de soporte social de los enfermos de TB. Este abordaje reflexivo objetiva mejorar la calidad de la investigación y la relevancia de los hallazgos para intervenciones más eficaces e inclusivas en el cuidado de la TB.

## Conclusión

Este estudio analizó cómo el control de la tuberculosis, caracterizado por un modelo jerarquizado y normativo, puede apagar la individualidad de los usuarios, tornando invisibles sentimientos como desamparo, angustia, aislamiento y miedo de la muerte. El abordaje del Programa Nacional de Control de la Tuberculosis, con su énfasis en manuales, técnicas y un lenguaje universal, contrasta con los cuidados producidos en la Atención Primaria a la Salud, que valoriza las singularidades de las realidades locales y los territorios vivos, en donde sucede la vida.

En las interpelaciones de la APS al PNCT se observa una disputa constante entre la política plural - que valoriza las diferencias y organiza las Redes de Atención a la Salud, de manera a garantizar el derecho democrático a la salud - y el modelo normativo del PNCT. La APS, con sus atributos, tiene por objetivo atender las singularidades de los usuarios, proponiendo un cuidado centrado en las necesidades individuales y en las especificidades de cada territorio.

Para fortalecer ese abordaje plural y democrático, es fundamental apoyar los espacios embrionarios de gobernanza, resultantes de la descentralización y regionalización de la salud. Esos espacios deben promover el protagonismo de los actores locales, vinculándolos a la pluralidad en el cuidado centrado en el usuario. Esa estrategia contribuirá para asegurar el derecho democrático a la salud de manera más asertiva, atendiendo a las necesidades específicas de los usuarios y promoviendo un cuidado más humano y eficaz.
